# Prevalence of skin picking (excoriation) disorder

**DOI:** 10.1016/j.jpsychires.2020.06.033

**Published:** 2020-11

**Authors:** Jon E. Grant, Samuel R. Chamberlain

**Affiliations:** a Department of Psychiatry & Behavioral Neuroscience University of Chicago, Chicago, IL, USA; b Department of Psychiatry, University of Cambridge & Cambridgeshire/Peterborough NHS Foundation Trust, UK

**Keywords:** Skin picking, Excoriation, Prevalence, Gender, Comorbidity

## Abstract

Skin picking (excoriation) disorder is a mental health condition characterized by repetitive picking of one's skin leading to tissue damage as well as functional impairment and/or distress. A convenience sampling of 10,169 adults, aged 18–69 years, representative of the general US population, completed a survey to establish occurrence of skin picking disorder. 213 participants (2.1%) (55.4% female) identified as having current skin picking disorder and 318 (3.1%) (54.1% female) reported lifetime skin picking disorder (i.e. current or past). Those with current skin picking disorder were significantly more likely to be female compared to those who never had skin picking (Likelihood Ratio, LR chi-square = 31.705, p < 0.001). Mental health comorbidities were common with generalized anxiety disorder (63.4%), depression (53.1%), and panic disorder (27.7%) being the most frequently endorsed. This study suggests that skin picking disorder is relatively common in the general population and typically characterized by high rates of comorbidity.

## 1 Introduction

Skin picking (excoriation) disorder is a psychological condition characterized by the repetitive and compulsive picking of skin, leading to tissue damage (American Psychiatric Association, APA, 2013). Even though discussed in the medical literature for over a century (Grant et al., 2012), skin picking disorder was not officially included as a mental health disorder in the APA's Diagnostic and Statistical Manual until the DSM-5 (2013).

To date, no nationwide epidemiological studies of skin picking disorder have been conducted, but there have been several smaller studies of the prevalence of skin picking disorder. The majority of the studies have been conducted in university settings. One recent electronic study examined the self-reported prevalence of past-month skin picking in college students (n = 4335) and found that 5.7% reported a clinical form of skin picking disorder (defined as occurring at least 5 times per day, causing physical damage e.g. skin lesions) and causing significant distress and/or functional impairment (Houghton et al., 2018). Another study, again conducted in students using an online survey, found that of the 1378 students completing the survey, 14% reported skin picking symptoms that indicated probable skin picking disorder (Solley and Turner, 2018). A study of 534 high school and university students in Poland, which used a self-report questionnaire based on the DSM-5, found that 5.43% of the total sample reported feeling significant distress caused by skin picking and 5.8% of the total sample reported functional impairment due to picking (Prochwicz et al., 2016). A study of 2196 Israeli university students used a 13-item self-report survey for skin picking and found that 3.03% reported symptoms consistent with skin picking disorder (Leibovici et al., 2014). Odlaug et al. (2013) surveyed 1916 university students and found a prevalence of 4.2% using questions based on the DSM-5 criteria. A study of 210 medical students (Siddiqui et al., 2012) found a probable prevalence rate of SPD of 9.0% (using criteria that a student must be involved in picking 5 times or more per day for 4 weeks or longer). A study of 439 students (again using a threshold of picking 5 times or more per day for 4 weeks or longer), found that 2.7% reported symptoms consistent with skin picking disorder (Teng et al., 2002). In a study of 245 university students in Turkey, 2.04% met criteria for skin picking on a self-report survey using criteria that mirrored the later DSM-5 criteria (Calikusu et al., 2012). In 2002, Bohne and colleagues studied 133 German students and reported a prevalence of 4.6% (skin picking defined as recurrent picking with significant impairment). To summarize, studies of university students have ranged in size from 245 to 4335, and have reported current prevalence rates ranging from 2.04% to 14%. It is unclear, however, whether these findings would generalize to the community at large.

A far smaller number of studies have been conducted in the community. In one study of 354 people randomly selected in public places, 63% of respondents engaged in some form of picking, but only 19 respondents (5.4%) reported significant picking with associated distress or impact (Hayes et al., 2009). Another study, based on 2,513 telephone interviews, found that 10% of respondents picked to the point of having noticeable skin damage that was not attributable to a medical condition (Keuthen et al., 2010). When distress or impairment was included as a diagnostic criterion, 1.4% met criteria for skin picking disorder. Finally, in a study in Brazil, 7639 participants were recruited using a web-based survey and a 12-item self-report form on skin picking. This study found that 3.4% endorsed current probable skin picking disorder (Machado et al., 2018).

The purpose of the current study was to fill these gaps in knowledge. We sought to determine the prevalence of skin picking disorder in a large representative sample from the US population. We were also interested in examining what reported comorbidities were most common in people with skin picking disorder, as well as the gender distribution of the illness.

## 2 Methods

### 2.1 Participants

The data collection was undertaken as part of market research for a client interested in providing a new treatment for trichotillomania. These data were then made available in de-identified form to the current researchers, who were free to interpret and publish the findings without any restrictions. Thus, the current paper constitutes a secondary analysis of de-identified data and was exempt from Institutional Review Board (IRB) procedures under current US guidelines. As part of the original data collection, all participants had provided informed consent and had agreed that their data could be shared in anonymized form with external researchers.

The Schlesinger Group, a well-known provider of panels for online surveys, used a convenience sampling method to enroll approximately 10,000 individuals in the general US population, ages 18–69 years. Quotas were used to assure a sample that was age and gender matched to the US Population (based on US Census Data). The Schlesinger Group is an ESOMAR member and adheres to the globally recognized code of conduct, the jointly developed ICC/ESOMAR Code, for marketing research. The procedures included a “double-opt-in” process for recruitment, requiring initial assent to the study participation policy, confirmation email being sent, and then the individual confirming assent by clicking an email link. This process of consent is standard for marketing research studies and is appropriate given the low level of risk to participants. The following quality control measures are used to validate the panel: photo ID validation (manual) at time of registration for panel; relevant ID and a programming (CAPTCHA) at registration to deter bots; a Red Herring survey to catch people outside of US, hidden questions in registration to catch bots, database checks to identify batches of similar email structure entering panel in short time period, profile checks to identify unlikely combinations of or too many combinations of ailments, and profile checks to identify selection of aberrant choices at different questions at registration and over time on the panel. The sponsor and all survey personnel were blinded to the identity of all survey subjects and had no access to any personally identifiable information. Schlesinger uses a point system for survey participants, and once a panelist obtains 500 points, they are able exchange it for rewards, with options to use it for Amazon dollars, gift cards with various retailers, or charities. The incentive for survey participation was 300 points, which has a value of $3.00. The survey took approximately 15 min to complete. The recruiting email for the survey did not make any mention of the focus of the survey on picking – no mention of “health”, “new treatment”, any diagnosis, or anything that could bias the survey in terms of those opting to participate.

### 2.2 Assessments

Each participant underwent an Internet-based, self-administered survey which included demographics and mental health history. The survey asked about multiple psychiatric disorders (skin picking disorder was included on the list) with one question (“Please indicate whether you currently have or have ever had any of the following medical conditions”). The survey was active from January 10 to January 24, 2019. The following quality assurance controls were used in the survey: non-leading survey invitation, to control for self-selection by panelists, a wide variety of diagnoses included in prevalence question to control for self-selection by panelists, check of survey participant responses vs their information at panel registration, check for speeding and/or straight-lining in survey responses, and active monitoring of survey patterns and removal of respondents who display inauthentic behaviors.

### 2.3 Data analysis

Data were presented descriptively. Where formal statistical tests were undertaken, these comprised independent sample t-tests for continuous variables, and Likelihood Ratio (LR) chi-square tests for categorical variables. Significance was defined as p < 0.05. Significance values are reported uncorrected.

## 3 Results

The study sample comprised 10,169 adults. The age, gender, education, race/ethnicity and annual household income of the screened sample of 10,169 adults mirrored that for the US population (U.S. Census Bureau, 2017, U.S. Census Bureau, 2018a, U.S. Census Bureau, 2018b). Table 1 shows an overview of the characteristics of the sample in each stratum.

**Table 1:** Demographics of the screened population and those with skin picking disorder.

Demographic Variables	Screened Population (n = 10,169)	Skin Picking Disorder, N and % of all SPD cases that fall into bracket	Prevalence of SPD in the stratum
**Age, years**			
18–29	2,551 (25.1%)	72 (33.8%)	2.82%
30–49	3,852 (37.9%)	89 (41.8%)	2.31%
50–69	3,766 (37.0%)	52 (24.4%)	1.38%
**Gender**			
Male	5,045 (49.6%)	87 (40.85%)	1.72%
Female	5,087 (50.0%)	118 (55.4%)	2.32%
Non-binary/third gender, not listed, or prefer not to answer	37 (0.4%)	8 (3.75%)	21.6%
**Education**			
High school diploma or less	1,564 (15.4%)	29 (13.6%)	1.85%
Some college or Associate degree	3,629 (35.7%)	56 (26.29%)	1.54%
Bachelor's degree	3,259 (32.1%)	55 (25.8%)	1.69%
Master's, Doctorate, or Professional degree	1,717 (16.9%)	73 (34.3%)	4.25%
**Race**			
American Indian or Alaska Native	185 (1.8%)	5 (2.35%)	2.70%
Asian	459 (4.5%)	11 (5.16%)	2.40%
Black or African American	1,853 (18.2%)	26 (12.21%)	1.40%
Native Hawaiian or Other Pacific Islander	49 (0.5%)	3 (1.41%)	6.12%
White	7,525 (74.0%)	179 (84.04%)	2.38%
Other	413 (4.1%)	2 (0.94%)	0.48%
**Annual Household Income**			
< $25,000	1,359 (13.4%)	45 (21.13%)	3.31%
$25,000 - $50,000	2,362 (23.2%)	42 (19.72%)	1.78%
$50,001 - $75,000	2,043 (20.1%)	40 (18.78%)	1.96%
$75,001 - $125,000	2,344 (23.1%)	50 (23.47%)	2.13%
> $125,000	1,596 (15.7%)	31 (14.55%)	1.94%
Prefer not to answer	465 (4.6%)	5 (2.35%)	1.08%

In total, 213 participants in the sample (2.1%) identified as having current skin picking disorder. Lifetime skin picking disorder (i.e. current or past) was reported by 318 participants (3.1%). The mean current age of those with skin picking disorder was 38.8 (standard deviation, SD, 13.8) years, and 118 (55.4%) of those with skin picking disorder were female. Those with current skin picking disorder were significantly more likely to be female compared to those who had never had skin picking (Likelihood Ratio, LR chi-square = 31.705, p < 0.001), and were also younger (age 38.8 [13.8] vs 43.5 [15.0], t = 4.531, p < 0.001). Those with skin picking disorder were more likely to report having White racial-ethnic affiliation (84.0% vs 73.8%, LR chi-square = 12.634, p < 0.001), but did not differ from those with no history of skin picking in terms of the highest level of education achieved (LR chi-square = 8.280, p = 0.218), nor current relationship status (e.g. single, cohabiting, or married) (LR chi-square = 6.752, p = 0.240).

The rates of current comorbidities reported in people with skin picking disorder were as follows (listed in order of decreasing frequency): generalized anxiety disorder (135 [63.4%]), depression (n = 133 [53.1%]), panic disorder (59 [27.7%]), post-traumatic stress disorder (PTSD) (58 [27.2%]), obsessive-compulsive disorder (OCD) (56 [26.3%]), attention-deficit hyperactivity disorder (ADHD) (50 [23.5%]), eating disorder (41 [19.3%]), drug or alcohol abuse (34 [16.0%]), trichotillomania (27 [12.7%]), bipolar disorder (26 [12.2%]), and tic disorder (15 [7.0%]).

## 3 Discussion

This study evaluated the prevalence of skin picking disorder, and its associations, in an epidemiologically representative community sample of 10,169 people. We found that skin picking disorder had a point prevalence of 2.1%, and lifetime prevalence of ~3.1%. These findings mirror the handful of previous community-recruited samples with much smaller sample sizes, which reported point prevalence of around 1.4% for skin picking disorder in one study, and 3.4% in another (Hayes et al., 2009; Machado et al., 2018). The variation in prevalence rates reported across studies could reflect natural variation expected statistically (i.e. repeatedly sampling prevalence from subsets of populations would yield a range around a mean); or could stem partly from differences in methodologies (e.g. the precise recruitment text used). The findings are important because they highlight that skin picking disorder is a prevalent mental health disorder. Yet in comparison to other disorders with similar prevalence, it has received very little research study in terms of neurobiology and treatments. For example, a PubMed search for [“skin picking” or “skin-picking”] and “treatment” yielded just 188 results; which contrasts to >7000 results for a similar search for obsessive-compulsive disorder, which has similar prevalence rates, and is the same DSM-5 diagnostic category (Kessler et al., 2005; Guo et al., 2016). In terms of neurobiology and cognition, a search yielded only 32 results for skin picking disorder, and >1000 for OCD.

In terms of gender distributions, the current study reported a slight preponderance of skin picking disorder in women versus men, including when compared to controls without the disorder. However, the gender differences in occurrence of the condition were not large – a ratio of 1.2 female:male. Most previous studies, mainly conducted in college students, have reported the disorder to be more common in women, albeit with considerable variation, with some studies reporting it to be relatively uncommon in men. The current results indicate that skin picking disorder should be considered as important in both men and women, since the gender distribution is not so different.

People with skin picking disorder reported extremely high rates of mental health comorbidities: the majority reported comorbid generalized anxiety disorder and/or depression. Around a quarter reported having panic disorder, PTSD, OCD, or ADHD (viewed individually). Eating disorders, substance use problems, trichotillomania, and bipolar disorder, were each reported by 10–20% of the sample; whereas tic disorders were reported by 7% of these individuals. As well as adding to the likely mental health burden of skin picking disorder, these findings also reinforce the importance of screening for mental health comorbidities in patients, and not only in the same diagnostic category. They also highlight that the concepts of impulsivity and compulsivity are important conceptually for skin picking disorder: impulsive and compulsive disorders were commonplace, as were mood and anxiety difficulties.

Several caveats should be noted. The study was based on anonymized unrestricted market research data provided to the authors. This limited the amount of information that was available to us. Research is needed to examine specific features of skin picking disorder in detail, such as in terms of age of onset, triggers, and phenomenology as well as cognitive function and neurobiology. The prevalence question was not framed in terms of whether or not the person has been formally diagnosed by a health professional with one of the mental health conditions, but was instead framed in terms of whether or not the person “has had” the condition. Additionally, this raises the related limitation that the survey did not assess for DSM-5 SPD criteria using items mapping onto individual criteria. The phrasing about having had a specific condition may also have inflated responses for co-occurring conditions. Although the findings were based on self-report rather than in-person clinical interviews, the advantage of the current approach was that a very large sample could be collected – this would simply not be possible with in-person assessments in this field. Also, while clinical assessments in-person are valuable and constitute the ‘gold standard’ for diagnosis, clinical findings often do not reflect the way disorders occur in the community at large, since they are conducted in rarefied samples (such as patients with more severe symptoms who are seeking treatment – most patients do not seek treatment, and most do not have severe symptoms).

In conclusion, this study demonstrated that skin picking disorder is a prevalent mental health condition with high rates of self-reported mood-anxiety, impulsive, and compulsive comorbidities, warranting much greater research and clinical attention.

## Author statement

All authors had access to the data. All made substantial contributions to the conception or design of the work as well as the acquisition, analysis, or interpretation of data; they all aided in drafting the work, gave final approval of the version to be published; and agree to be accountable for all aspects of the work in ensuring that questions related to the accuracy or integrity of any part of the work are appropriately investigated and resolved.

## Declaration of competing interest

The researchers’ time for this study was funded by internal funds. The survey data were collected by Promentis Pharmaceuticals, Inc., and were made available for unrestricted use by the study authors.

The authors received no funding from Promentis Pharmaceuticals, Inc., for this study. Promentis Pharmaceuticals, Inc., has had no influence on the analyses of data or the writing of this manuscript. Dr. Grant has received research grants from TLC Foundation, and Otsuka Pharmaceuticals. Dr. Grant receives yearly compensation from Springer Publishing for acting as Editor-in-Chief of the Journal of Gambling Studies and has received royalties from Oxford University Press, American Psychiatric Publishing, Inc., Norton Press, and McGraw Hill. Dr. Chamberlain's time on this study was supported in part by a Wellcome Trust Clinical Fellowship (110049/Z/15/Z). Dr. Chamberlain consults for Promentis Pharmaceuticals, Inc., on work unrelated to this manuscript, and Ieso Digital Health. Dr. Chamberlain receives a stipend for his work as Associate Editor at Neuroscience and Biobehavioral Reviews; and at Comprehensive Psychiatry.
